# The importance of setting and therapeutic relationships when delivering chiropractic care to those living with disadvantage

**DOI:** 10.1186/s12998-022-00456-y

**Published:** 2022-10-23

**Authors:** Dan Marthick-Hone, Aunty Kerrie Doyle, Gerard A. Kennedy, Dein Vindigni, Barbara I. Polus

**Affiliations:** 1 Discipline of Chiropractic, School of Health and Biomedical Sciences, RMIT University, Bundoora, Australia; 2 Indigenous Health School of Medicine, University of Western Sydney, Campbelltown, Australia; 3 Institute of Health and Wellbeing, Federation University, Ballarat, Australia; 4 School of Engineering, RMIT University, Bundoora, Australia

**Keywords:** Chiropractic, Patient-centred care, Patient reported outcome measures, Musculoskeletal pain, Disadvantaged communities

## Abstract

**Background:**

Chiropractic is a mostly privatised health profession within Australia, with people experiencing disadvantage typically having limited access due to financial barriers. However, some universities within Australia offer community outreach clinics where students provide chiropractic care to people living with disadvantage. This demographic experiences higher rates of chronic conditions including musculoskeletal complaints and requires subsidisation to access privatised care. This need also offers opportunity for the chiropractic profession to work within community healthcare teams. A mixed-methods observational study was used to investigate how the unique setting of a student chiropractic community clinic may influence the experience and outcomes of those who attend.

**Methods:**

Three patient-reported outcome measures (PROMs) investigated client outcomes: Measure Yourself Medical Outcome Profile (MYMOP); European Five Domain Five Level Quality of Life Questionnaire (EQ-5D-5L); and the Patient Enablement Instrument. The PROMs data were analysed descriptively and inferentially. Interviews were conducted with clients who had received chiropractic care, chiropractic students, clinical supervisors and staff of the centre. Interview data were coded using thematic analysis, and themes were formed using Bronfenbrenner’s socio-ecological systems framework and non-participant observations.

**Results:**

Thirty-seven participants completed baseline PROMs and 17 completed follow-ups after four treatments. Seventy-two percent of participants nominated their primary complaint as chronic. Significant change was noted in general health and wellbeing for the MYMOP, pain and disability for the EQ-5D-5L and index scores for the EQ-5D-5L suggested improved health and wellbeing. Most clients experienced higher levels of enablement post treatment. Twelve participants were interviewed (four were clients), with five themes emerging from the interview data. Clients reported their lived experiences impacted their health problems and attending the clinic offered benefits beyond improvement of pain and disability.

**Conclusions:**

Interview data suggested that these benefits were due to a combination of therapy, the setting and the relationships formed within that setting. Complementing this, PROM data suggested clients experienced better levels of health and wellbeing and decreased levels of pain and disability. Findings indicated that people who experienced disadvantage may receive broader benefits from attending community centres offering chiropractic care. Services such as chiropractic may be complementary in meeting the healthcare needs of those experiencing disadvantage.

## Background

Disadvantage can have significant negative influence on the health of those who experience it in their lives [1–3]. In healthcare, disadvantage is typically defined as any factor that may influence, or lead to, poorer health outcomes than one’s peers [4]. Disadvantage may present in numerous ways. Some examples include homelessness and rough-sleeping, lower socioeconomic or education status, unemployment, or underemployment status and those marginalised by race or age [5–9]. Globally, those who live with, or experience disadvantage, suffer from higher rates of chronic conditions and comorbidities [10–13]. In relation to chronic low back pain, there is a strong association between its prevalence and socioeconomic status, in high-income and low-income countries alike [14]. It is also known that the management of chronic low back pain through interventions such as surgery and opioid prescription is typically ineffective for this condition [14]. This limits treatment options for those experiencing disadvantage and multiple multimorbidities, as most allied health care is privatised and expensive to access for these groups [15, 16]. Alongside higher rates of chronic conditions and comorbidities, those from lower socioeconomic backgrounds may also experience discrimination in the healthcare system, potentially leading to avoidance behaviours when seeking healthcare [17–20].

Patient-centred care (PCC), while defined in various ways, is a paradigm of holistic healthcare that recognises each patient as an individual with unique physiological and biopsychosocial needs [21]. This includes tailoring the consultation to the patient, developing treatment goals, building a relationship of trust and negotiating with the patient to develop their care plan [22]. When delivered appropriately, PCC aims to inform and involve the patient in their own healthcare in a way that leads to better outcomes [23]. The outcomes associated with PCC have been extensively investigated with the aim of improving patient care and cost-effectiveness of interventions [21, 24, 25]. This is achieved by using patient-reported outcome measures (PROMs) [26]. Patient-reported outcome measures allow a unique assessment of patients’ individual outcomes, health and wellbeing and quality of life. Patient-reported outcome measures can be either disease-specific or more generic, with the goal of measuring outcomes that are of importance to the patient completing them. Generic outcome measures have been used to assess treatment modalities including traditional Chinese medicine (acupuncture) [27], chiropractic [28], physiotherapy [29] spinal surgery [30], and while disease specific outcome measures have addressed conditions such as chronic low back pain [31], mental health disorders [32] and lumbar scoliosis [33]. While PROMs have been widely utilised, little investigation has reported on PROM usage for those who experience both musculoskeletal health conditions and disadvantage, including whether MSK health outcomes improved or not, and how factors, such as setting, patient-practitioner relationship, and patient-centred care, might influence these outcomes. For this reason, a combination of PROMs and interviews were used in this investigation to better understand the experiences of those who attended and delivered care at the chiropractic clinic within The Wellington (TW).

**Fig. 1:**
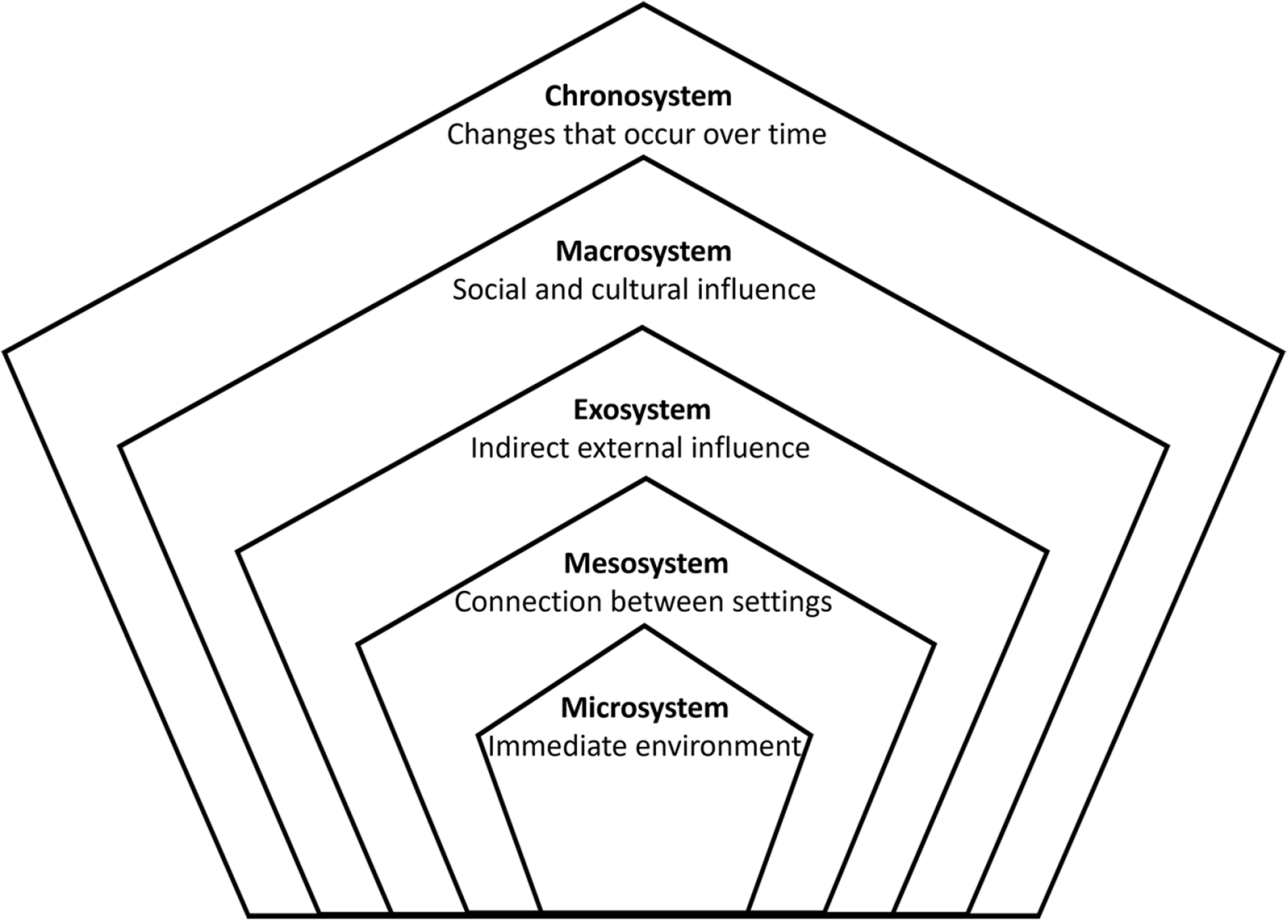
Bronfenbrenner’s Systems, adapted from Bronfenbrenner, 1977 [34]

In this study, the socio-ecological systems framework of Bronfenbrenner was used in the thematic analysis of interview data [34]. This framework states that the development of any individual is influenced by five systems that comprise an individual’s ecosystem (Fig. 1). These five systems are described as: (1) the microsystem, a person’s immediate settings in which they participate; (2) the mesosystem, a collection of microsystems within a person’s life that influence each other; (3) the exosystem, external settings that the individual is not involved in but is influenced by; (4) the macrosystem, the overarching influence of a society’s beliefs, religions, laws and morals; and (5) the chronosystem, how the passage of time may influence a person’s life, either past, present or future [34, 35].

The socio-ecological systems framework discusses the importance of a setting and its impact on the development of the people within it [34–36]. Bronfenbrenner’s framework proposes that if a setting has indirect links to other settings of power, then this can directly influence the potential for development of the person involved in those settings. Bronfenbrenner’s ecosystems framework describes how the greater the indirect links a setting such as this might have, the greater its developmental potential [35]. The Wellington is an example of a setting that provides developmental potential for its clients by offering holistic services addressing health, social and wellbeing dimensions of peoples’ lives.

### Aim

The aim of this study was to investigate, using mixed methods, the importance of setting and its influence on outcomes, when delivering chiropractic care for people experiencing musculoskeletal pain and living with disadvantage. This study focused on clients, staff and students delivering chiropractic care within a community centre known as TW.

## Methods

This mixed-methods observational study utilised a combination of PROMs, semi-structured interviews, and non-participant observation. The research design was based on an emerging framework of research known as Whole Systems Research [37]. This framework integrates quantitative and qualitative research methods. Whole Systems Research has been previously used to assess allied health and complementary and alternative medicine (CAM) because these therapies tend to be wholistic in their approach to patient care [37–40].

### Location and demographics

The Wellington was located at 215 Wellington Street, Collingwood, Victoria, Australia. This investigation followed clients who attended TW for the student chiropractic clinic, operated by Royal Melbourne Institute of Technology (RMIT) University. The location of this clinic was later moved, during the data collection period, in September of 2019, to level 1 of the Melbourne Polytechnic building. This new location was only a few hundred metres away from the old location at 20 Otter Street, Collingwood, Victoria, Australia. The Wellington was first established in 2004 and historically operated from St. Joseph’s Church and then later St. Martin’s Community Church. The Wellington offers a place of community, inclusion and friendship in a welcoming atmosphere that aims to offer support and assistance to those suffering from disadvantage, marginalisation and social and cultural isolation within the community [41]. The Wellington has numerous partnerships with community organisations and institutions including local universities (that offer allied health and CAM therapies), hospitals, churches, and the City of Yarra Council. Although TW provides other forms of healthcare, the focus of this study was on the delivery of chiropractic care. When the clinic operated at St Martin’s it would transform during clinic days into an open plan clinical setting with curtained partitions for client privacy. The pews were placed against the back wall to allow room for the chiropractic interns to place six to eight portable tables along the walls for treatment. These tables would then be partitioned to allow for client privacy. This clinical space was utilised by the different clinical services offered within TW throughout the week on different days. The RMIT chiropractic interns were all within their final clinical years of completing their degree (fourth or fifth year). The shifts that students attended were on a rotational basis and the chiropractic clinic was open on Wednesday and Friday afternoons from 1:30 pm to 4:00 pm. This meant that continuity of care with the same practitioner was sometimes difficult to maintain for the client.

When TW moved physical location, more space was allocated for patients with eight treatment rooms permanently established, each with their own individual treatment tables, chairs, and desks. Each treatment room included a door and frosted glass, providing client privacy during consultations.

### Recruitment

Participants for this study were all recruited from within TW. All new attendees of the chiropractic clinics at TW were initially informed of the study via the welcoming receptionists and a flyer that contained information regarding the study was available at the reception desk for clients to read if they wanted to learn more about the research project. If a client of the clinic was interested in participating in the study, the receptionists would provide them with a participant information sheet that described the study and what participation involved. Alternatively, upon the client`s request, the receptionist introduced the potential participant to the researcher who was in attendance whenever the chiropractic clinic was operating. Before any data were collected from participants, written informed consent was obtained.

### Participants

This study included three participant groups, with the focus being clients who attended the RMIT student chiropractic clinic located at TW. Participant groups were as follows:

- Clients attending the chiropractic clinic at TW;
- Fourth and fifth year RMIT chiropractic interns who were treating clients at TW; as well as their clinical supervisors; and
- Staff of TW (manager and receptionist).

All participants in this study were over the age of 18 years with the ability to give informed written consent.

All participant information was de-identified and a unique code was assigned to their data. Clients were given the option to complete PROMs in private or with the investigator (DMH) if assistance was needed. Patient-reported outcome measures were completed at baseline before treatment, and after their fourth visit or after two weeks (whichever came first). All interviews were conducted in private. The investigator, at no time, had access to the health records kept by the clinic at TW. The student chiropractor informed the client when it was their fourth visit to the clinic (or after two weeks) and asked the client if they would like to complete follow up questionnaires with the investigator. If the client responded that they were willing to complete follow up questionnaires, the investigator (DMH) was notified. This study was approved by the Human Research Ethics Committee of RMIT University. Approval Number 21684. Data collection took place from February 2019 until December 2019.

### Inclusion and exclusion criteria

The inclusion criteria for clients (Table 1) in this study who completed PROMs included anyone presenting with a self-selected complaint. The participants were all either new to the clinic or existing clients of the clinic who presented with a new complaint that required assessment. This approach was used to minimise external factors that might contribute to, or influence, a change in the condition (particularly if it was already being treated elsewhere). Clients who were already having their primary complaint treated elsewhere were not eligible for participation. Throughout the course of the study, participants were also asked to disclose if they had received any secondary treatment from any of the other services offered within TW clinic. This was performed at collection of follow up data. This was performed to minimise external factors that might have interfered with participant outcomes. If participants did receive treatment elsewhere during their chiropractic treatment schedule, they were omitted from further follow-up data collection. If a participant’s condition required referral to a medical practitioner, they were also omitted from the study.

**Table 1:** Inclusion/exclusion criteria for clients who completed PROMs

Inclusion criteria	Exclusion criteria
New clients presenting to the RMIT student chiropractic clinic at TW	A client who had a new complaint but was already having this managed externally to TW regardless of healthcare provider
An existing chiropractic client at TW who had a new complaint that had not been assessed or treated by the students interning at TW. Conversely, someone who had been a client of TW chiropractic clinic but had returned after a six-month + absence and required a full review before treatment commenced	A participant of the study who disclosed they received care from another provider at TW, or externally (Chinese medicine, myotherapy, osteopathy, general practitioner, physiotherapist, or other healthcare professional)
Over the age of 18 with the ability to give informed consent to participate in the study	Under the age of 18, unable to provide informed consent or unable to read and understand the participant information and consent forms

All clients who fulfilled these inclusion criteria were followed over a course of treatment. Treatment was delivered by RMIT University chiropractic interns in their fourth or fifth (final) year of study. Participants presented with a wide variety of conditions, and each received a combination of treatment modalities forming an individual treatment plan for, depending on their diagnoses.

The inclusion criteria for clients who were interviewed were: (1) over the age of 18; (2) able to give informed written consent; and (3) had been a patient of the RMIT chiropractic clinic at TW in the past.

The inclusion criteria for chiropractic interns, the clinical supervisor, the manager and staff/volunteers of TW was that they were either on placement at TW clinic or were staff/volunteers at TW and were physically present at the time when the chiropractic clinic was operating.

### Chiropractic care at The Wellington

Chiropractic utilises a wide range of treatment modalities in order to affect an individualised treatment for the client and their presenting musculoskeletal (MSK) complaint. The modalities used by the RMIT chiropractic interns at TW included various combinations of spinal manipulative therapy (SMT) which utilises a high velocity low amplitude thrust to manipulate joints of the body [42], soft tissue therapies (STT) [43], hand-held adjusting instruments such as an activator [44] and mechanical wedges (blocks). These mechanically assisted instruments are often used as an alternative treatment method for SMT as they can provide a low-force method of spinal mobilisation. Students at TW also prescribed home care and exercises for clients that could be completed between visits. This care was delivered by one chiropractic intern per patient, under the supervision of an Australian Health Practitioner Regulation Agency (AHPRA) registered chiropractor [45].

### Patient-reported outcome measures

Three PROMs were chosen for use in this investigation. They were selected based on their reliability, validity, and ability to detect change across time. The first PROM was the Measure Yourself Medical Outcome Profile (MYMOP). The MYMOP was originally developed with the aim of allowing the respondent to nominate symptoms of their primary health complaint and an activity in their day-to-day lives impacted upon because of that symptom [46]. This was important, as it allowed the respondent to choose symptoms and an activity that was specifically important to them, rather than choosing from a list of options. The MYMOP has been used in several studies in the past. Some of these include the evaluation of patients receiving chiropractic treatment [47], traditional Chinese medicine for long-term conditions [27] and inpatients suffering from acute exacerbations of chronic bronchitis [48]. The MYMOP also asks the participant to list any medication they are taking and how important it is that they reduce or avoid taking medication if they are not currently taking any. The MYMOP was deemed appropriate for use in this study given its usefulness in measuring change in chronic conditions and its measurement of patient-specific individual outcomes.

The second PROM was the European Quality of Life Five Domain Five Level Health Questionnaire (EQ-5D-5L). The EQ-5D-5L is a health-related quality of life outcome measure that asks the respondent to nominate, on a five point scale, how good or bad their health is pertaining to five different domains of health [49]. These domains are “Mobility”, “Personal Care”, “Usual Activities”, “Pain and Discomfort” and “Anxiety and Depression”. Accompanying these domains is a 100-point visual analogue scale known as the European Quality of Life Visual Analogue Scale (EQVAS). The EQVAS asks the respondent to nominate on this scale how good or bad their health had been that day. The EQ-5D-5L has been used in a number of studies investigating outcomes of patients with chronic conditions [50], knee osteoarthritis, chronic osteoarthritis, low back pain or cancer pain [51] and to determine the cost-effectiveness of care received [52]. The EQ-5D-5L was chosen over the first version (EQ-5D-3L) due to its higher sensitivity to change [53]. It was also chosen as a suitable adjunct to the MYMOP. Because responses from the MYMOP are individual and based on nominated symptoms and activities, it was deemed important to also have a tool that measured outcomes that were the same for all participants.

Baseline EQ-5D-5L profile scores were used to calculate index values which is a single number that reflects how good or bad a respondent’s health was. These index scores are based on value sets provided by the EuroQol Research Foundation and are only available for some countries [54]. A value set for Australia is not currently available and so the value set for the United Kingdom was used instead to determine index scores for this study. The United Kingdom value sets were used to determine this study’s index scores due to the similarity of their health and vitality indices according to the Australian Institute of Health and Welfare [55].

The third measure was the Patient Enablement Instrument (PEI). The PEI is a PROM that measures the enablement of patients as a result of attending a health service [56]. Enablement is considered as empowerment of the patient and their ability to comprehend, understand and cope with their particular illness or condition [57]. The PEI asks the respondent six questions pertaining to their consultations over the past weeks or months. The PEI has been used within the literature to assess the enablement of patients suffering from chronic musculoskeletal pain [58]. In addition, as this was an observational study, it was important to include a PROM that asked respondents questions regarding outcomes that specifically pertained to the treatment they received as opposed to external factors.

The MYMOP and EQ-5D-5L were administered at baseline to all clients who agreed to participate in the study. At follow-up the MYMOP and EQ-5D-5L were again administered, this time together with the PEI.

All data from the MYMOP and EQ-5D-5L were analysed using paired-sample t-tests where data were normally distributed. Where data were not normally distributed, Wilcoxon signed-rank tests were used. Data from the PEI underwent descriptive analysis only.

### Interviews

Clients, student chiropractors, chiropractic supervisors and staff of TW were invited to participate in interviews. Interviewing groups other than clients assisted in determining how different components of chiropractic care at TW were considered to benefit clients from the carers’ perspective, what other factors may have influenced client outcomes and to what extent TW, as a setting, may have influenced these outcomes.

Interviews were semi-structured in their design and pre-determined prompts were utilised where necessary. There were two sets of interview questions used, one for clients and one for all other participant groups. The interview schedule for clients contained questions such as “*Please tell me something about your health problem and how it affects you*” and *“How did you come to attend this clinic for it?”.* The interview schedules for the other participant groups included questions such as “*Can you tell me about your first impressions of starting work at The Wellington?”* and *“… what effects, if any, has working in this clinic had on you?”.*

All interviews were conducted, audio recorded and transcribed verbatim by the chief investigator (DMH) at TW, in private. Using Braun and Clarke’s method for thematic analysis, six steps were followed: (1) familiarisation; (2) coding; (3) generating themes; (4) reviewing themes; (5) defining and naming themes; and (6) writing up themes with supporting statements. This process was undertaken by DMH and repeated (DMH & AKD). Emerging inconsistencies were addressed (BP). To ensure familiarisation with the data, interviews were relistened to and transcripts were reread. Coding was conducted manually, using a spreadsheet. During coding, the main content of what was being said was highlighted throughout all the transcripts. Once initial coding was completed for all transcripts, the codes were then grouped into larger categories that emerged from the data. A constant comparative approach was used, where interview data were analysed and coded after each interview [59]. Interviews were conducted until data saturation occurred (no new information was being discovered in the analysis of interview data) [60]. All transcripts were returned to the respective interviewees, where possible, to ensure verification of accuracy.

Urie Bronfenbrenner’s Socio-ecological systems framework was applied in a thematic analysis of the interview data [61, 62]. Codes and voices were matched to Bronfenbrenner’s specific Social systems using the Socio-ecological framework. Using this framework, codes were developed into themes that then reflected the micro- to macro-systems [61].

Non-participant observation was used to gain a deeper understanding of the interview findings [63]. It provided an opportunity to describe the different interactions that took place in the common area/waiting room between those at TW who sought chiropractic care and to allow for a deeper understanding of other qualitative and quantitative findings. Non-participant observational self-reflections of the researcher (DMH) were used to provide additional data for categorising codes into themes in harmony with the socio-ecological systems framework [35]. These reflections were not written at the time of observation, but later during the same day, and were very general in their description, to protect the anonymity of clients at TW.

## Results

A total of 37 clients completed PROMs at baseline. Follow-up was possible with 17 of these clients. Of those 37 clients, 16 were male and 21 were female. The average age of clients was 52 years. Of those who completed follow-up for the MYMOP 11 were female and 6 were male, with a mean age of 58. Data are forthwith referred to as mean ± standard deviation.

### Measure yourself medical outcome profile

**Fig. 2:**
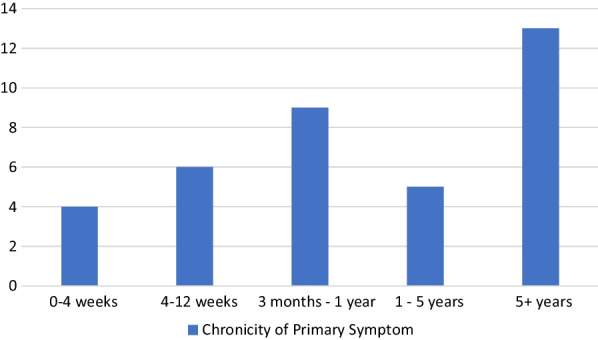
Chronicity of Primary Symptom—bars indicate frequency of reported chronicity of primary symptom

**Fig. 3:**
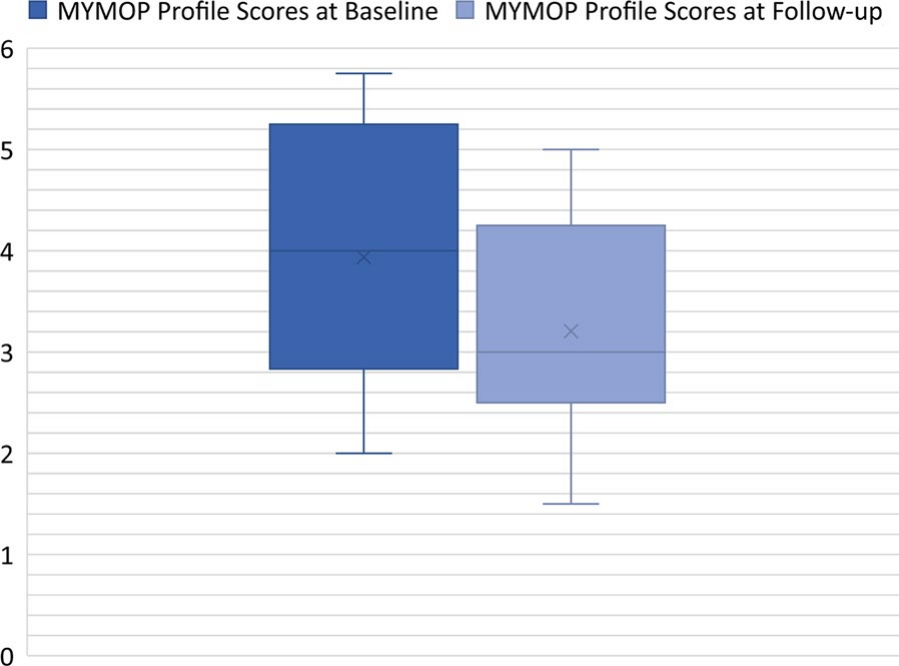
MYMOP Profiles Scores at Baseline and Follow-up N = 17. Measure Yourself Medical Outcome Profile Scores at Baseline and Follow-up. The box represents 95% C.I.; solid lines within the boxes are median values; X refers to mean values; whiskers refer to maximum and minimum values

At baseline, 72% participants reported attending TW for a problem that was chronic in nature (see Fig. 2) and 75% of clients stated that they were not currently taking any medication to assist with their primary symptom. For this study, chronicity was defined as having lived with the symptom for 3 months or longer. More than half of the clients in this study stated that avoiding medication usage for their symptoms was of some importance to them. Significant change was detected between baseline (3.94 ± 1.22) and follow up (3.2 ± 1), [*t*(16) = 2.84], (*p* = 0.012) MYMOP profile scores (see Fig. 3).

### European five doman five level quality of life questionnaire

The assumption of normality for the five domains of the EQ-5D-5L was not satisfied for any of the domains, as assessed by the Shapiro–Wilk test (*p* < 0.05). Wilcoxon signed-rank tests showed no significant differences between baseline and follow-up for the EQ-5D-5L domains of “Mobility” (*p* = 0.589), “Personal Care” (*p* = 0.414), “Usual Activities” (*p* = 0.314) and “Anxiety and Depression” (*p* = 0.608). However, a statistically significant change was noted for the domain of pain and discomfort (*p* = 0.014).

**Fig. 4:**
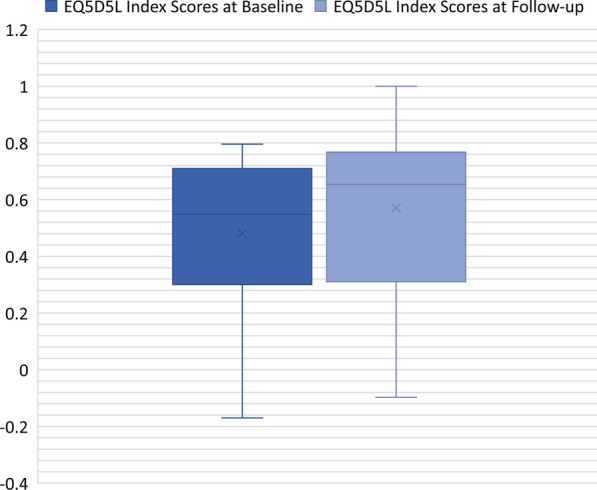
Index Scores at Baseline and Follow-up n = 17. European Quality of Life Five Domain Five Level Index Scores at baseline and follow-up. The box represents 95% C.I.; solid lines within the boxes are median values; X refers to mean values; whiskers refer to maximum and minimum values

A paired-samples t-test was used to determine if there was a statistically significant change between baseline (0.48 ± 0.27) and follow-up (0.57 ± 0.30) EQ-5D-5L index scores. Data were normally distributed, as assessed by Shapiro–Wilk test (*p* > 0.05). There was a statistically significant change noted in baseline and follow-up EQ-5D-5L index score [*t*(16) =  − 2.3], (*p* = 0.033) (Fig. 4).

### Patient enablement instrument

Seventeen participants completed the PEI at follow-up. While not all clients who completed the PEI experienced improved scores pertaining to enablement, the majority did. (Table 2).

**Table 2:** Patient enablement instrument responses n = 17

Question	Much better/more n (%)	Better/more n (%)	Same or less n (%)
Able to cope with life	5 (29.4)	6 (35.3)	6 (35.3)
Able to understand your illness	6 (35.3)	8 (47.1)	3 (17.6)
Able to cope with your illness	7 (41.2)	7 (41.2)	3 (17.6)
Able to keep healthy	4 (23.5)	6 (35.3)	7 (41.2)
Confident about your health	6 (35.3)	6 (35.3)	5 (29.4)
Able to help yourself	6 (35.3)	9 (52.9)	2 (11.8)

### Interview findings

Twelve interviews were conducted with participants, four clients, three chiropractic students, three chiropractic supervisors and two staff at TW. From the interview findings, codes were grouped into themes using Bronfenbrenner’s Socio-ecological systems framework [35]. This study was only able to obtain interviews from pre-existing clients at TW. A total of five themes were developed, each in relation to one of Bronfenbrenner’s five systems. Non-participant observations assisted with the development of these themes and codes and allowed for greater context during the analysis of interview data.

#### A patient-centred approach for clients

The first theme was titled *A Patient-Centred Approach for Clients* and was developed in relation to what is known as the microsystem (Table 3). This theme comprised three codes that were related to how clients experienced chiropractic care at TW, how they were treated at TW and how TW acted as the primary setting for this study. The first code was *The Client Experience at The Wellington*. This code was synthesised from comments made by participants regarding the delivery of chiropractic care by students and the way in which this type of care was delivered. These comments were all related to events, relationships and interactions that took place because of RMIT University’s and TW’s agreement to offer chiropractic to the clients of TW and the education that students receive at RMIT University.

“It’s, been interesting learning curve. I mean, you guys that talk in a different language to me and I mean, I’ve found out a lot more about how my own body functions compared, cos, I basically knew nothing before”—Client of TW.

**Table 3:** Themes and descriptions

Ecosystem	Theme	Summary of theme
Microsystem	A patient-centred approach for clients	A space offering positive social interaction and support for the clients who attend TW
Mesosystem	Clients are complex	Mobility and function are important to clients as it enables them to participate in the activities of daily living
Exosystem	Trust	Meaningful relationships of trust were formed between clients of TW and RMIT chiropractic students
Macrosystem	The cost of being disadvantaged	Pain management and physical mobility remained the primary focus for clients with chronic conditions
Chronosystem	Looking to the future	The chiropractic service at TW has been available at low or no cost to disadvantaged members of the Collingwood community since 2004. Several clients depend on this care to get through their daily lives with minimal discomfort

The second code was *Client Treatment Protocol at The Wellington*. Interview findings suggested that students try and use a patient-centred approach when treating clients at TW. This was identified in comments made by clients, student practitioners and clinical supervisors alike. It was also found that clients typically preferred a lower-force approach to their treatment that involved education, home-exercise, lifestyle advice, hand-held chiropractic adjusting devices and soft tissue therapies. This code indicated that clients were involved in the development of their management plans across time and that therapeutic relationships were formed. This highlighted that while the student practitioners were developing their skills in delivering patient-centred care, they were certainly receptive to feedback from the clients at TW.

“I tell ‘em what my problems are and if they aren’t manipulating strong enough, or they’re not hitting the right spots. I tell them and they’re appreciative of any feedback they get and I think they need that feedback because otherwise you’re just treating people like lining up at a, at a queue in Centrelink, you’re just a number”—Client of TW.

The third code in this theme was *The Wellington as a Setting.* The Wellington was identified as an important service for the community. It offered a place for individuals within the community to congregate and form meaningful and genuine relationships with others, helping battle the social isolation some clients may experience. It was also identified as an important learning experience for students who treated clients at TW.

“I know mental health’s a lot of old blokes like myself think it’s a bit of a wank, but the mental health issue is having someone to talk to and the community environment was really helpful.”—Client of TW.

The findings associated with this theme demonstrated that clients attending TW for chiropractic care experienced benefits beyond improvements in pain and disability. Clients described that the clinic offered hope for their future and how their mental health had improved because of attending. It was also reported that clients who attended the chiropractic clinic at TW experienced PCC from the students who treated and cared for them. The Wellington was described as important for the clients who attended this setting. Consistent with the description on the TW’s website, that it `encouraged meeting and interacting with others who attend`, interviewed participants also discussed how TW as a setting provided a sense of community and an opportunity to simply chat with someone.

#### Clients are complex

The first code in this theme was *Benefits and Reasons for Attending Chiropractic at The Wellington.* The maintenance or improvement of physical mobility and function to complete activities was found to be of importance to clients. These findings were individual to each patient. Clients continued to return to TW at high rates according to staff. It was reported that not all clients may have received improvement in their presenting health problems, according to their patient files.

“Well, just keep maintaining it or maybe eventually it might get a little bit better.”—Client of TW.

The second code was *Impact of Health Problems on Clients’ Lives.* Clients discussed how their individual health problems impaired their ability to participate in their daily activities. Clients also discussed how, in certain circumstances, they needed to plan for the future, as their health conditions were chronic. One client was aware of the consequences of their condition and had to make preparations surrounding their work-related activities.

“That’s ah, that’s difficult. Uhm, getting out of bed’s been difficult.”—Client of TW.

The third code in this theme was *Chronicity and Multiple Conditions.* Clients reported having chronic conditions. Supervisors, students, and clinical supervisors reported that some clients presented with not only physical conditions but also mental health problems. It was also reported that clients might present with more than one condition and that they sometimes had complex medical and personal health histories.

“Ah, physically and mentally, you know, a lot of things going on in their life and/or their bodies that make their treatment in some ways, a bit more challenging…”—Chiropractic Student at TW.

#### Trust

The third theme was titled *Trust* and was based upon what Bronfenbrenner described as the exosystem (Table 3). This theme was produced from codes surrounding the relationships clients formed with those at TW, students and their delivery of care and clients’ experience with external healthcare providers. In the first code, *Relationships,* interview respondents discussed how important trust was as a key component of forming and maintaining genuine relationships with the clients at TW. Clients described how they appreciated the care they received at TW and how they particularly valued students who were genuine in their approach to caring for clients.

“So, it’s been really good. Not just for the treatment side of things, but also the kids that the, uhm, the students, ah, are affable, they’re, they’re talkative.”—Client of TW.

The second code was *Student Delivery of Chiropractic Care at The Wellington.* Clients acknowledged that they did prefer some students over others. However, they stated how they were happy with the care they received from all students. Clients preferred students who were genuine in their approach to treatment and communication and preferred to be treated as an individual and not a number. Clients made bonds with their student practitioners over short periods of time and were understanding that students needed to move on for various reasons.

“…you can see that they’re thinking about what they’re doing and they’re making good choices.”—Client of TW.

In the third code, *Client Experience with External Healthcare Providers,* clients described how they perceived the care they received at TW as being separate from healthcare delivered/provided outside this setting, although clients acknowledged how healthcare providers work together in serving the betterment of clients’ health as their common goal. While most clients did not report discrimination, one client did report that they had experienced discrimination from other healthcare providers external to TW in the past.

“Uhm, they’re, they’re happy with it, as long as I’m happy. If I get the benefit out of it, even if it’s just a small token.”—Client of TW

#### The cost of being disadvantaged

The fourth theme was titled *The Cost of Being Disadvantaged* and was formed using Bronfenbrenner’s macrosystem (Table 3). This theme was constructed by two codes surrounding *Clients Experience with Medication* and the financial barriers they faced when trying to access primary healthcare such as chiropractic at TW. In the first code, similar to the findings from the PROMs, all clients reported that they preferred to avoid taking medication for their complaint, primarily because they did not find their usage effective in managing their symptoms for an extended period of time. While they did prefer to avoid medications, several clients were still using them for a range of conditions, including anxiety and depression.

“But now since I’m on anti-depressants, uhm, and my social anxiety’s largely gone away, uhm, I find it a lot easier, just to be part of society.”—Client of TW.

Clients also reported some frustration that services like TW were not more widely available. In the second code, *Financial Barriers Impair Access to Chiropractic Care,* they described that healthcare such as chiropractic should be available to a wider population, regardless of income status. Clients also reported that they were appreciative of home care and advice that was provided by students, as this enabled them to manage their complaints until their next appointment.

“…it just offers such a wonderful, wonderful opportunity for people to get the treatment.”—Client of TW.

#### Looking to the future

The final theme was titled *Looking to the Future* and was generated using what Bronfenbrenner described as the chronosystem (Table 3). This theme comprised codes that described how TW was an important setting for those who live within the local community, how it acted as a place that addressed their healthcare needs and how the clients were hopeful regarding the future of their health conditions. Also, that they planned to continue using TW as a place to address these needs.

The first code in this theme was *The Wellington as a Setting Over Time.* In this code clients reported how they relied on TW and that they hope for it to continue as a community service for years to come. It was stated that the change in location was seen as a positive for clients and staff described how the move would allow for an expansion of services such as the chiropractic offered there. Students only offered minimal responses regarding change they wanted to see at TW, such as better hygiene practices and free parking.

“Yeah. To me it’s really just, yeah. Expanding on services that we really have. Now, not really re-inventing the wheel, but just, yeah, finding the time and the space to do more, for more people.”—Staff Member at TW.

In the second code, *Future of Clients and Their Healthcare Needs,* Clients stated that they were positive regarding the future of their health conditions. Clients expressed how they understood their chronic conditions might never resolve. However, they were determined to continue seeking care as they found value in maintaining the current state of their condition or at least maintaining current levels so that their condition did not deteriorate.

“If I get back, well, health-wise not too good. But we’ll get there. But If I can get back into having treatment here in say the next, I’m hoping next 6 weeks. Then, then I’ll feel as if I’m on the road to recovery.”—Client of TW.

## Discussion

Prominent findings of this investigation suggest that clients who attended TW for chiropractic care experienced high rates of chronicity related to their health problems and improved levels of health and wellbeing and pain and discomfort after only a short schedule of care at the clinic. Clients reported experiencing improved levels of enablement as a result of attending the clinic. Interview data revealed that outcomes of care that clients found important to them were improvement and maintenance of physical mobility and function and mental health and wellbeing. It was also reported that meaningful relationships of trust were formed at TW. This indicated that the outcomes that clients experienced may be due to a combination of therapy, the setting in which care is delivered and the relationships and bonds that are formed within that setting.

The literature suggests that those who experience disadvantage have higher rates of chronic conditions and comorbidities when compared with the general population [64–66]. This study’s findings aligned with the literature, as a vast majority of the clients nominated having had their primary symptom (as nominated on the MYMOP) for greater than three months in duration. This was also iterated in the findings with all clients interviewed expressing how they lived with and managed their chronic conditions.

Data from the MYMOP indicated that many of the clients preferred to avoid using medication to manage their symptoms. Some studies indicate that it is common for people experiencing chronic MSK conditions, or socioeconomic disadvantage, to also have long-term opioid prescriptions to manage their pain [67, 68]. Interview findings from the four client participants suggested that the reason for this low rate of medication usage among the clients at TW was because they found the usage of pain medication to be ineffective in the management of their symptoms. Other studies suggest that the usage of medications such as non-steroidal anti-inflammatories for chronic low back pain offer minimal benefits and that opioid prescriptions for chronic non-cancer pain may lead to poorer levels of function in the individual taking them [69, 70]. Interviews and PROMs did not, however, investigate the usage of self-prescribed drugs, which is common in those experiencing disadvantage [71]. Some evidence does suggest that people experiencing chronic conditions who receive CAM therapy reduce medication usage [72]. Of those who did use medication for the management of symptoms, the majority described that it was of some importance to them that they reduce medication usage where possible.

While this study had a relatively small sample size, with less than 50% of participants being able to provide follow-up data, the results of the from the MYMOP and EQ-5D-5L indicated that clients experienced a statistically significant improvement in their health and wellbeing after a short course of treatment at TW. The literature suggests that those with complex care needs require a combination of both healthcare and social care [73, 74], and the outcomes of interviews at TW were congruent with these findings. The social aspects of attending TW were clearly of great importance to clients, with some clients even suggesting that this social interaction improved their mental health and gave them hope for the future.

Another pertinent finding of this study was that favourable levels of enablement were experienced by the clients who attended TW, as captured by the PEI. The literature suggests that those who experience disadvantage also tend to have decreased levels of enablement [75, 76]. The findings of favourable levels of enablement are positive and were validated by the interview data. Clients expressed that because of their treatments they were better able to manage their conditions even when students were absent from the clinic. It was also reported that clients valued being given home exercise and advice for when their conditions had acute exacerbations and they had to wait until their next consultation.

Clients described the importance of improved or maintained levels of physical mobility and function. Clients also described that because of their care at TW they were better able to complete their individual daily tasks. The social components of attending TW and being able to receive care simultaneously were particularly important for the health of the clients of TW. This finding is consistent with the literature which describes an association between those experiencing social isolation and disadvantage, and having decreased levels of physical mobility, particularly in older adults [77]. These findings may indicate that the social components of attending TW may, in part, influence these outcomes.

### Limitations

This study had several limitations. The small sample size of PROM participants and follow up data comprising less than 50% of initial participants must be considered as a limitation to this study, as does the small number of interviews (four) conducted with existing clients of TW. Follow-up with clients only occurred after a short timeframe, with eligibility restricted to clients who had received four treatments or two weeks after their first visit and there was no further follow-up. This made it difficult to determine if the benefits of attending the clinic were long-lasting or only temporary, although, to some extent, interview data (where participants were long-term clients of TW) suggested that benefits were long-lasting. It is important not to assume that benefits experienced by clients were a direct result of chiropractic treatment. Other factors may have influenced these outcomes such as natural history of a condition, acute exacerbations of a chronic condition or the influence of TW setting, as mentioned previously. There may have been some unavoidable inherent bias by respondents as there could have been an expectation to provide favourable responses in a setting they clearly appreciated, depended upon, and where they felt welcomed. Another limitation is that client interviewees were not new clients to the clinic, interview findings suggested they were long-standing clients who continued to return to the clinic due to the benefit they personally received. This may account for some discrepancies between the quantitative and qualitative findings. However, this did allow for a deeper exploration into the aspects of care, such as a sense of community and the setting, as these clients had greater opportunity to experience this. It is also important to consider that any outcomes observed in this study may have been influenced by the placebo effect, or some other contextual influence that was unknown to the research team.

### Recommendations for future research

While the clients who were interviewed in this investigation were all long-term clients of TW, PROM data revealed the majority of those who attended TW throughout the data collection period were a transient population. This meant that many clients did not return for subsequent visits and were unable to complete follow-up outcome measures as a result. Future research should therefore allow more time for data collection to take place. It may be appropriate to separate the interviews into different groups for analysis to achieve a deeper understanding of experience. This would also mean interviewing each group until no new codes or themes emerged, rather than all interviewees as a single group. To increase sample size and data collection, it is also recommended that the inclusion criteria be expanded. In this instance TW offered other complementary health services such as osteopathy and traditional Chinese medicine. To include clients who used these services would have resulted in a larger data sample. Similarly, if there are other settings that offer comparable services to those in need it would, where possible, be advisable to include them in data collection. This would allow for a comparison between settings and to see if outcomes may differ as a result. This study was only able to obtain interviews from existing clients at TW and while they were able to provide rich answers, it would also be valuable to see how new clients experience TW and to see if these responses differed.

## Conclusion

The findings of this investigation indicated that TW as a setting, and the chiropractic clinic operating within it, may provide a nurturing space for those living with disadvantage to experience meaningful physical and psychosocial health outcomes. Outcomes important to the clients of TW varied on an individual basis, although analysis assisted in identifying codes and themes surrounding these outcomes. Genuine relationships with their practitioners, the maintenance and improvement of their symptoms and benefits associated with mental health were all outcomes that were identified as important to the client. Patient-reported outcome measure data supported these findings, with improved scores of wellbeing noted in MYMOP scores and in the EQ-5D-5L index scores. A key component of enablement requires a healthy therapeutic relationship between the practitioner and patient (Mercer et al., 2008). Given the high level of enablement reported by clients of TW, as demonstrated by PEI scores, it is reasonable to assume that the genuine relationships experienced by interview participants are also occurring between new clients to the clinic and their student practitioners.

Finally, findings from this study indicate TW as a setting provides a gathering space which enables clients to interact in a supportive, welcoming setting as an integral part of the treatment they receive. Beyond the reported improvements in physical mobility and reduction in pain it also enables the development of friendships and nurtures a sense of belonging and community. All the groups interviewed as part of this study expressed how important TW is as a place for those experiencing disadvantage as it offers a safe space with many resources, including chiropractic care. This presents an opportunity for the chiropractic profession, one that could potentially see the profession starting to become a part of the conversation surrounding the healthcare needs of those living with disadvantage.

## Data Availability

The datasets generated and/or analysed during the current study are not publicly available due to reasons of sensitivity surrounding individual human data but are available from the corresponding author on request. Data are being stored under controlled access at RMIT University.

## Abbreviations

EQ-5D-5L: European quality of life five domain five level health questionnaire
EQVAS: European quality of life visual analogue scale
MSK: Musculoskeletal
MYMOP: Measure yourself medical outcome profile
PCC: Patient-centred care
PEI: Patient enablement instrument
PROMs: Patient-reported outcome measures
TW: The Wellington
